# Honokiol inhibits *in vitro* and *in vivo* growth of oral squamous cell carcinoma through induction of apoptosis, cell cycle arrest and autophagy

**DOI:** 10.1111/jcmm.13474

**Published:** 2018-01-24

**Authors:** Kao‐Jean Huang, Chin‐Ho Kuo, Shu‐Hsin Chen, Ching‐Yen Lin, Ying‐Ray Lee

**Affiliations:** ^1^ Development Center for Biotechnology Institute of Biologics New Taipei City Taiwan; ^2^ Division of Hematology‐Oncology and Blood Bank Department of Internal Medicine Ditmanson Medical Foundation Chia‐Yi Christian Hospital Chiayi Taiwan; ^3^ Department of Medical Research Ditmanson Medical Foundation Chia‐Yi Christian Hospital Chiayi Taiwan; ^4^ Department of Nursing Min‐Hwei College of Health Care Management Tainan Taiwan

**Keywords:** Honokiol, cell cycle arrest, apoptosis, autophagy, human oral squamous cell carcinoma

## Abstract

Honokiol, an active natural product derived from *Magnolia officinalis*, exerted anticancer effects through a variety of mechanisms on multiple types of cancers. In this study, the molecular mechanisms of honokiol in suppressing the human oral squamous cell carcinoma (OSCC) cells were evaluated. Treatment of two OSCC cell lines with honokiol resulted in reducing the cell proliferation and arresting the cell cycle at G1 stage which was correlated with the down‐regulation of Cdk2 and Cdk4 and the up‐regulation of cell cycle suppressors, p21 and p27. In addition, the caspase‐dependent programmed cell death was substantially detected, and the autophagy was induced as the autophagosome formation and autophagic flux proceeded. Modulation of autophagy by autophagic inducer, rapamycin or inhibitors, 3‐MA or bafilomycin, potentiated the honokiol‐mediated anti‐OSCC effects where honokiol exerted multiple actions in suppression of MAPK pathway and regulation of Akt/mTOR or AMPK pathways. As compared to clinical therapeutic agent, 5‐FU, honokiol exhibited more potent activity against OSCC cells and synergistically enhanced the cytotoxic effect of 5‐FU. Furthermore, orally administrated honokiol exerted effective antitumour activity *in vivo* in OSCC‐xenografted mice. Thus, this study revealed that honokiol could be a promising candidate in preventing human OSCCs.

## Introduction

Oral squamous cell carcinoma (OSCC) is one of the most common neoplasia worldwide 1, 2, which is often diagnosed in the oropharynx and oral cavity. It is highly invasive and metastatic at the advanced stage, and presents a substantial threat to human health 2, 3. In Taiwan, the incidence and mortality of oral cancer in males ranked fourth in the list of top 10 male cancers 3. Besides cigarette and alcohol, betel quid chewing is proved to be tightly associated with the carcinogenesis of oral cancers in Taiwan and in South‐East Asia 4, 5. To date, current treatment of oral caners involves surgery or radiation in combination with chemotherapy using chemicals, such as 5‐Fu and cisplatin 2, 6, 7. However, surgery and radiation treatments inevitably cause negative impacts on patients’ appearance and oral functions such as chewing and speaking. Furthermore, these treatments are not satisfied as the 5‐year and 10‐year relative survival rates are less than 30% 2, 8 as the OSCC is being usually diagnosed at late stage. Thus, it is urgent to develop an efficient and safer antitumour agent against OSCC in clinical use.

Honokiol, a biphenolic phytochemical, is one of dominant biphenolic compounds isolated from Magnolia plant species. It has been used in Traditional Chinese Medicine (TCM) for the treatment of various biological properties including antidepressant, anti‐emetic, anti‐oxidative, antithrombotic, anti‐angiogenesis, anti‐anxiolytic, anti‐inflammatory and antitumour activities 9, 10, 11. Moreover, it also has been found to exert potent activities against the broad spectrum of various micro‐organisms, including bacteria, viruses and fungi 11, 12, 13, 14, 15, 16. *In vitro* and *in vivo* studies suggest that honokiol has multiple anticancer actions against numerous solid and haematological cancers including human hepatoma, myelogenous leukaemia, lung adenocarcinoma, breast cancer, ovarian cancer, prostate cancer and gastrointestinal cancer 11, 17, 18, 19, 20, 21, 22, 23.

Recently, honokiol alone (in comparison with 5‐FU) has been shown to inhibit the cell growth of two OSCC cell lines *in vitro*, HSC‐3 and HSC‐4, through the induction of apoptosis 24, but other anticancer mechanisms exerted by honokiol on OSCC cells have not yet been fully studied, especially when the OSCC cells were originated from different stimuli, that is betel quid. Here, we investigate the anticancer mechanisms of honokiol on another two OSCC cell lines that derived from two males with habits of drinking, smoking and betel quid chewing in Taiwan, and conclude that honokiol exerted multiple actions against these two cell lines by arresting cell cycle at G1 phase, inducing apoptosis, regulating cell signalling pathway and, in particular, provoking autophagy.

## Materials and methods

### Cell lines and cell culture

Two OSCC cell lines (OC2 and OCSL), derived from males with habits of drinking, smoking and betel quid chewing in Taiwan 25, were maintained with RPMI1640 medium (FBS; Gibco, Grand Island, NY, USA) supplemented with 10% foetal bovine serum (FBS; Gibco) and 1% penicillin/streptomycin (Gibco). The other cell line, SAS, was purchased from Japanese Collection of Research Bioresources Cell Bank (JCRB) and was maintained with Dulbecco's modified Eagle's medium (DMEM; Gibco) supplemented with 10% FBS and 1% penicillin/streptomycin. Cells were cultured at 37°C supplied with 5% CO_2_. In addition, SAS cells were transfected with siRNA (purchased from Ambion, Austin, TX, USA) to block the expression of ATG5. Lipofectamine 2000 (Invitrogen, Rockville, MD, USA) was mixed into serum‐free DMEM containing siRNA or scrambled RNA.

### Cells viability analysis

Honokiol was purchased from BioVision (Mountain View, CA, USA). Cells (5 × 10^3^ cells/100 μl) were plated into 96‐well tissue culture plates and grown in the above‐mentioned medium. The cells were treated with medium only (containing 0.01% DMSO) or medium containing honokiol at 5, 10, 20, 30, 40, 50 and 60 μM. After cultivation for 24, 48 and 72 hr, the number of metabolically active cells was determined by cell counting kit‐8 (CCK‐8) assay (Enzo Life Sciences, Farmingdale, NY, USA). The final results were analysed by statistical methods in three independent studies.

### Cell cycle analysis

Cells were incubated with either honokiol as indicated dosages or DMSO 0.01% for 24 or 48 hr. Cells were harvested and fixed with 70% ethanol overnight. After washing with PBS, cells were labelled with 500 μl PI staining buffer (Sigma‐Aldrich, St. Louis, MO) and incubated at room temperature in the dark for 30 min. DNA content was analysed using FACScan (Becton Dickinson, San Diego, CA) with ModFit LT 3.3 software. In addition, the cell cycle markers, such as cyclin D1, cyclin E, cdk4, cdk2, p21 and p27, were determined by Western blot with the antibodies (Cell Signaling, Danvers, MA, USA).

### Analysis of cell apoptosis by annexin V/PI staining

After treatment of the cells, annexin V staining (Sigma‐Aldrich) was performed to detect apoptotic cells. The cells were washed with PBS twice and centrifuged at 1500 × *g* for 10 min. The cell pellets were resuspended in 100 μl of staining solution (2 μl annexin V‐FITC and 2 μl PI in 100 μl binding buffer) and incubated for 15 min. at room temperature in darkness. Annexin V or PI fluorescent intensities were analysed by FACScan (Becton Dickinson, San Diego, CA), and 10,000 cells were evaluated in each sample.

### Western blot analysis

Cells were cultured in 10‐cm culture dishes and treated with or without compounds. The DMSO was used as negative control. The cell lysate was prepared using lysis buffer (50 mM Tris‐Hcl pH 7.5, 1 mM EDTA, 150 NaCl, 50 mM NaF, 1 mM Na_3_VO_4_, 1 mM PMSF, 1% NP‐40)with cocktail of protease inhibitors, and the protein concentration in the supernatant was determined by Bio‐Rad protein assay kit (Bio‐Rad, Hercules, CA, USA). The cell lysate (50 μg) was subjected to the sodium dodecyl sulphate–polyacrylamide gel electrophoresis (SDS‐PAGE) analysis, and the separated proteins were electrically transferred to a PVDF membrane (Millipore Corporation, Bedford, MA, USA). The membrane was probed with sequential additions of primary and matched secondary antibodies, and the signal was developed with enhanced chemiluminescence (ECL) substrate and acquired by BioSpectrum 800 Imaging System (UVP, CA, USA). The antibodies used were as follows: anti‐LC3 (Abgent, San Diego, CA, USA), anti‐Akt (Santa Cruz Biotechnology, Santa Cruz, CA, USA), anti‐phosphorylated‐Akt (Santa Cruz Biotechnology), anti‐mTOR (Cell Signaling), anti‐phosphorylated‐mTOR (Cell Signaling), anti‐phosphorylated‐p70S6 K (Cell Signaling), anti‐p62 (Abgent), anticyclin D1 (Cell Signaling), anti‐Cdk4 (Cell Signaling), anti‐p27 (Cell Signaling), anticyclin E (Cell Signaling), anti‐Cdk2 (Cell Signaling), anti‐p21 (Cell Signaling), anti‐caspase‐8 (Cell Signaling), anti‐caspase‐3 (Cell Signaling), anti‐PARP (Cell Signaling), anti‐Bcl‐xl (Epitomics, Burlingame, CA, USA), anti‐Bid (Epitomics), anti‐caspase‐9 (Cell Signaling), anti‐GAPDH (GeneTex, San Antonio, Texas, USA) and secondary antibodies, antimouse IgG Ab and anti‐rabbit‐IgG Ab(GeneTex).

### Immunofluorescent staining

Cells (1 × 10^5^ cells/well) were seeded on a slide and cultured for 24 hr. The cells were treated with DMSO, rapamycin or honokiol, and then fixed in methanol for 20 min. The slides were incubated for 30 min. in 0.1% Triton X‐100 in phosphate‐buffered saline. Anti‐LC3 polyclonal antibody (Medical & Biological Laboratories, Naka‐ku, Nagoya, Japan) was added on the slide and left overnight at 4°C. The fluorescent change in the cells was captured and analysed by high‐content image analyzer, BD pathway 435 (BD Biosciences, San Jose, CA). The percentage of cells with LC3 puncta formation and the average number of LC3 puncta per cell were analysed by BD Attovision software (BD Biosciences).

### *In vivo* anticancer assay (xenograft nude mice model)

The 6‐week‐old male nude mice (BALB/cAnN.Cg‐Foxn1nu/CrlNarl) were purchased from the National Laboratory Animal Center. Mice were maintained in the animal facility in the Department of Life Science, National Dong Hwa University, Hualien, Taiwan. OC2, OCSL and SAS cells (2 × 10^6^ cells/mice) were separately injected subcutaneously (s.c.) into the right flank of the nude mice, but only the SAS xenograft nude mice model was successfully established. The experiment protocol was conducted as follows: Six‐week‐old male nude mice were randomized into three groups (three mice/group in each experiment); 2 × 10^6^ cells of SAS were injected s.c. at day 0. After 10 days post‐injection, and the tumour was growth at less 1 mm^3^, mice were treated with DMSO or honokiol. Control group mice were orally fed with DMSO, and the experimental group mice were orally fed with honokiol 5 mg/kg or 15 mg/kg at days 1, 4, 7, 10, 13, 16, 19 and 22, Tumour size was measured at above days. Tumour volume was estimated by the equation (*L* × *S* ^2^/2, *L* as the longest diameter, *S* as the shortest diameter). Closing the experiment on day 35, the nude mice were killed, and the tumour was harvested and weighed. Two independent experiments were conducted. The experimental protocol was complied with Taiwan's Animal Protection Act and was approved by the Laboratory Animal Care and Use Committee of the National Chiayi University (IACUC Approval No. 104016).

### Statistical analysis

Data are presented as mean ± S.D. for the indicated number of separate experiments. Differences between the test and control groups were analysed by the one‐way anova and Fisher's least significant difference test. In the *in vivo* mice study, the Mann–Whitney *U*‐test was used. Statistical significance was defined as a *P*‐value less than 0.05 in all tests.

## Results

### Honokiol suppresses the growth of OSCC cells

**Figure 1:**
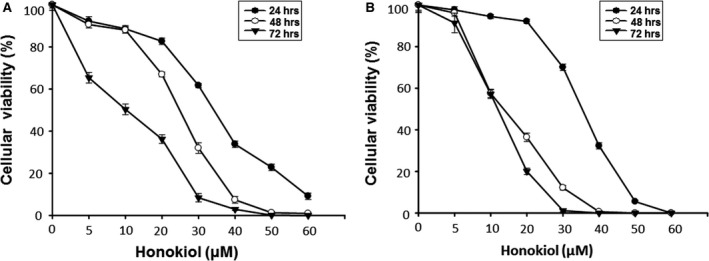
Honokiol inhibited the growth of human OSCC cell lines. Cells were incubated without or with various concentrations of honokiol for 24, 48 and 72 hr, and the cellular viability of (**A**) OC2 and (**B**) OCSL cells was analysed by the CCK‐8 assay. All of the data were expressed as mean ± S.D. of three independent experiments.

Human OSCC cell lines, OC2 and OCSL, were used to examine the potential effect of honokiol. The growth inhibitions of OC2 and OCSL cells were observed in a dosage‐dependent and time‐dependent manner after honokiol treatment (Fig. 1). The effect of honokiol on the growth inhibition was evident, and the GI50 for OC2 and OCSL was 35 and 33 μM and 22 and 13 μM at 24 and 48 hr, respectively (Fig. 1). The OCSL cells with malignant phenotype were more sensitive to honokiol treatment than OC2 cells (Fig. 1). A selective cytotoxicity of honokiol on tumour and normal cells was differentiated by the treatment of Hs68, a normal human fibroblast cell line, where the GI_50_ of honokiol on Hs68 cells was 70 μM and 43 μM at 24 and 48 hr (Fig. S1), respectively. The data elevated a therapeutic potentiality of honokiol for human OSCC cells.

### Honokiol arrests the progress of cell cycle at G0/G1 phase

**Figure 2:**
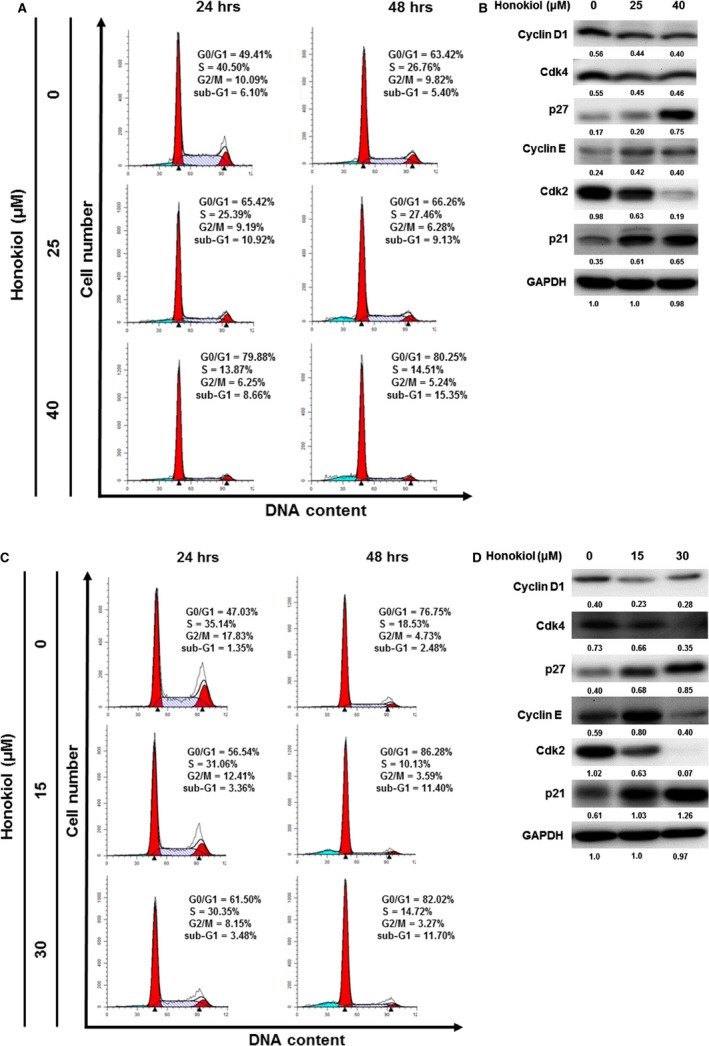
Honokiol arrested the progress of cell cycle at G0/G1 phase in the human OSCC cell lines. (**A**) OC2 and (**C**) OCSL cells were incubated with various concentrations of honokiol for 24 and 48 hr, and the cell cycle stages were determined by flow cytometric analysis. The expression of cyclin D1, Cdk4, p27, cyclin E, Cdk2 and p21 in the (**B**) OC2 and (**D**) OCSL cells was analysed by Western blotting, and GAPDH was used as an internal control.

As the growth inhibition of OSCC cells exerted by honokiol was obvious, we next examine whether the cell cycle of OSCC cells treated with various doses of honokiol was affected. By flow cytometric analysis, OC2 and OCSL cells showed their cell cycle progression was arrested mostly at the G0/G1 phase accompanied by the gradually reduced S and G2/M phases in a dose‐ and time‐dependent manner (Fig. 2A and C). In support of this, proteins involved in regulating cell cycle progression were examined by Western blotting. Treatment of honokiol resulted in the accumulation of cyclin E and the up‐regulation of cell cycle inhibitors, p21 and p27, but decreased the expression of Cdk2 (Fig. 2B and D). In addition, the expressions of cyclin D1 and CDK4 in OC2 and OCSL cells were decreased under honokiol treatment (Fig. 2B and D). Taken together, these results supported that honokiol caused cell cycle arrest notably at G0/G1 phase. Moreover, honokiol also slightly increased the sub‐G1 population at 48 hr (Fig. 2A and C), suggesting that honokiol may exert the cytotoxic effect at later stage.

### Honokiol induces cell death through apoptosis pathway

**Figure 3:**
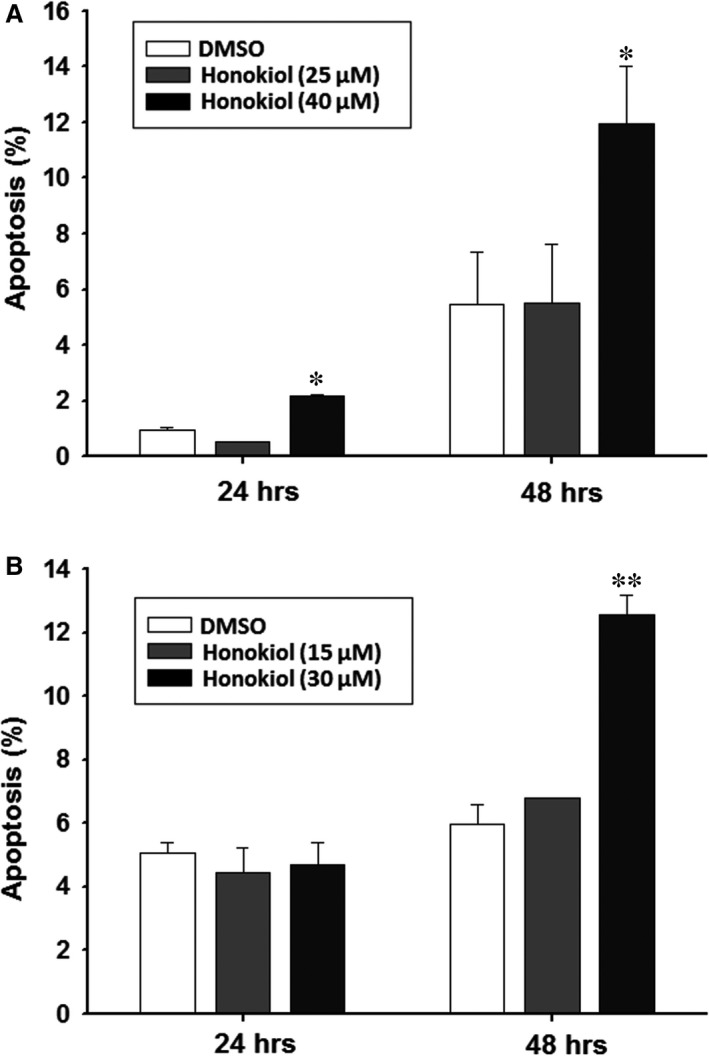
Honokiol‐induced apoptosis in human OSCC cells. (**A**) OC2 and (**B**) OCSL cells incubated with honokiol for 24 and 48 hr were harvested and stained with PI/annexin V for flow cytometry. The percentage of apoptotic cells were calculated and plotted. The data present as the mean ± S.D. of three independent experiments. **P < 0.01.

**Figure 4:**
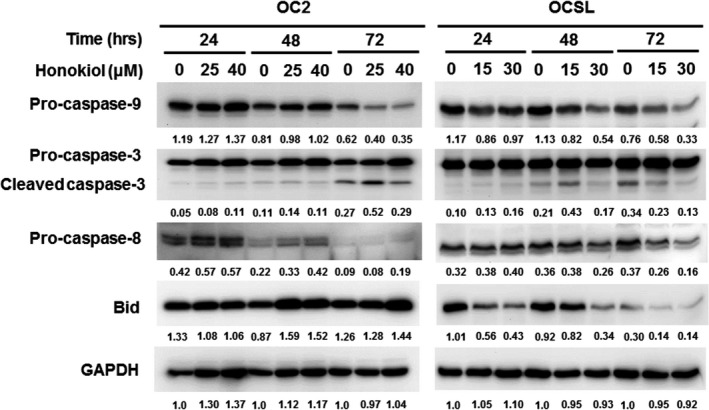
Detection of honokiol‐mediated apoptosis in human OSCC cells. OC2 and OCSL cells treated with various concentrations of honokiol for 24, 48 and 72 hr, and the expression of caspase‐9, caspase‐8, Bid and cleavage forms of caspase‐3 was analysed by Western blotting. GAPDH was used as an internal control.

To monitor the apoptosis induced by honokiol, OSCC cells were assayed using annexin V‐FITC and PI staining. Treatment of honokiol at low dose (i.e. 25 μM for OC2 and 15 μM for OCSL cells) did not increase the population of annexin V^+^/PI^+^ cells at 24 and 48 hr, but mount a slight effect on the increase in apoptotic population (from 5% to 12% of OCSL cells) at 48 hr and at higher dose (i.e. 40 μM for OC2 and 30 μM for OCSL) (Fig. 3). Although the apoptosis induced by honokiol was not prominent by flow cytometry at early stage (24 hr for OC2 cells and OCSL cells), the involving signal transduction pathway was proceeded as the cleaved caspase‐3 was appeared at 48 hr of OCSL cells and at 72 hr of OC2 cells (Fig. 4). These two cell lines are different intrinsically in response to honokiol treatment. In OC2 cells, the levels of full‐length form of caspase‐9 were slightly decreased at 72 hr post‐treatment, which was accompanied by the elevation of cleaved form of caspase‐3. No significant change was observed in caspase‐8 expression between non‐treatment and treatment groups (Fig. 4). However, in OCSL cells, treatment of honokiol resulted in the decreased expression of caspase‐8 and caspase‐9 as well as Bid protein, a Bcl family member (Fig. 4), suggesting the heterogeneity of OSCC cell origins varied in their responses to honokiol treatment. These data demonstrated that caspase‐dependent apoptosis was partially involved (not a major effect) in the honokiol‐mediated anticancer ability.

### Honokiol induces autophagy

**Figure 5:**
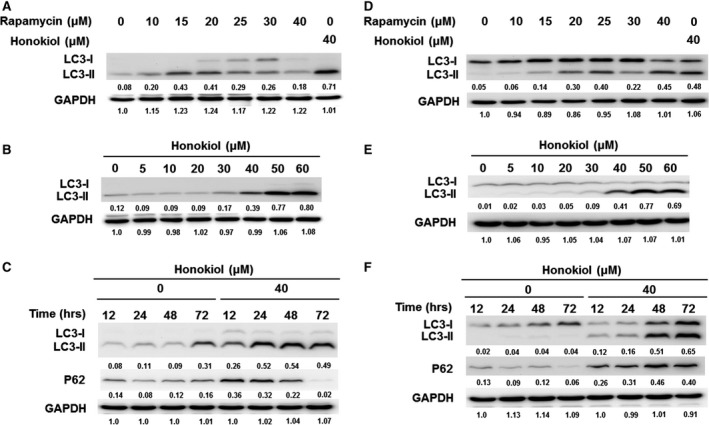
Autophagy induced by honokiol in human OSCC cells. To clarify that honokiol induced autophagosome formation, an inducer (rapamycin) of autophagy was tested. The expression levels of LC3‐II were determined for various concentrations of rapamycin and honokiol treatment in (**A**,** B**) OC2 and (**D**,** E**) OCSL cells. (**C**) OC2 and (**F**) OCSL cells were treated with honokiol (0 and 40 μM), and then, the cell lysates were collected at 12, 24, 48 and 72 hr for Western blotting of p62 proteins. GAPDH was used as an internal control.

**Figure 6:**
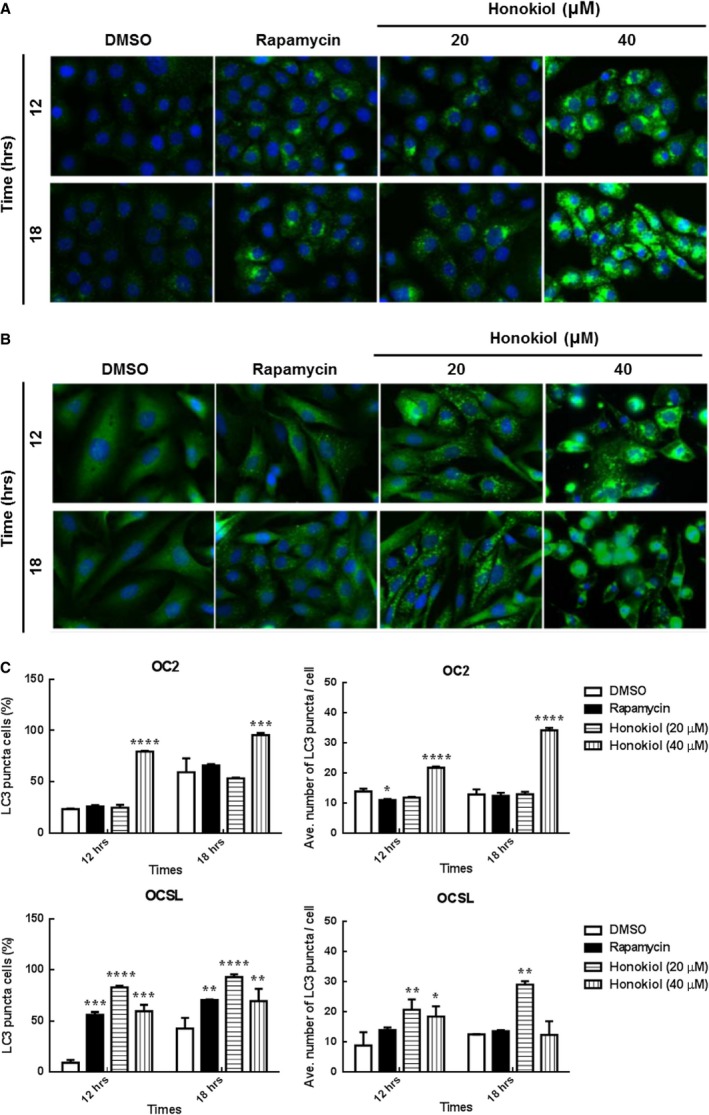
Autophagy was triggered after honokiol treatment in human OSCC cells. (**A**) OC2 and (**B**) OCSL cells were treated with DMSO, rapamycin and honokiol for 12 and 18 hr. Cells were stained with anti‐rabbit LC3, and the autophagosome was determined. (**C**) High‐content image analysis of LC3 puncta in (**A**) and (**B**) was determined, respectively. The fluorescence intensity of LC3 was analysed by BD Attovision software. The data present as the mean ± S.D. of three independent experiments. *P < 0.05, **P < 0.01, ***P < 0.001, ****P < 0.0001 *versus* DMSO.

To clarify the ability of honokiol‐induced cell death in human OSCC cells extensively, autophagy was examined in honokiol‐treated OSCC cells. Although the basal level expression pattern of LC3 (the marker of autophagy) in OC2 and OCSL cells under normal condition was different, that is OC2 cells showing endogenous LC3‐II expression, the expression levels of LC3‐II post‐honokiol treatment in OC2 and OCSL were comparable to those of rapamycin treatment in each cell line (Fig. 5A and D). The data showed that honokiol induced a stronger autophagy than rapamycin in both OC2 and OCSL cells (Fig. 5A and D). Honokiol induced autophagy in a dosage‐dependent manner (Fig. 5B and E), and one marker of autophagy, LC3‐II, could be detected as early as 12 hr post‐treatment (Fig. 5C and F). Moreover, the autophagic flux proceeded following the autophagy induction as the p62 expression was increased along with the autophagy process (Fig. 5C and F). By comparison between OC2 and OCSL cells, the effect of honokiol on LC3‐II expression in OCSL cells was more obvious. In support of this, OSCC cells treated with honokiol were stained with anti‐LC3 antibody in immunofluorescence assay and represented the puncta pattern of LC3‐II aggregation (Fig. 6A–C). Cotreatment with the autophagy inhibitor, 3‐MA, attenuated honokiol‐induced LC3‐II expression in both OC2 and OCSL cells (Figs. S2B and S3B). Taken together, these data demonstrated that honokiol triggered autophagy and autophagic flux in human OSCC cells.

### Honokiol deregulates the Akt/mTOR signalling pathway and may activate the AMPK signalling molecule

**Figure 7:**
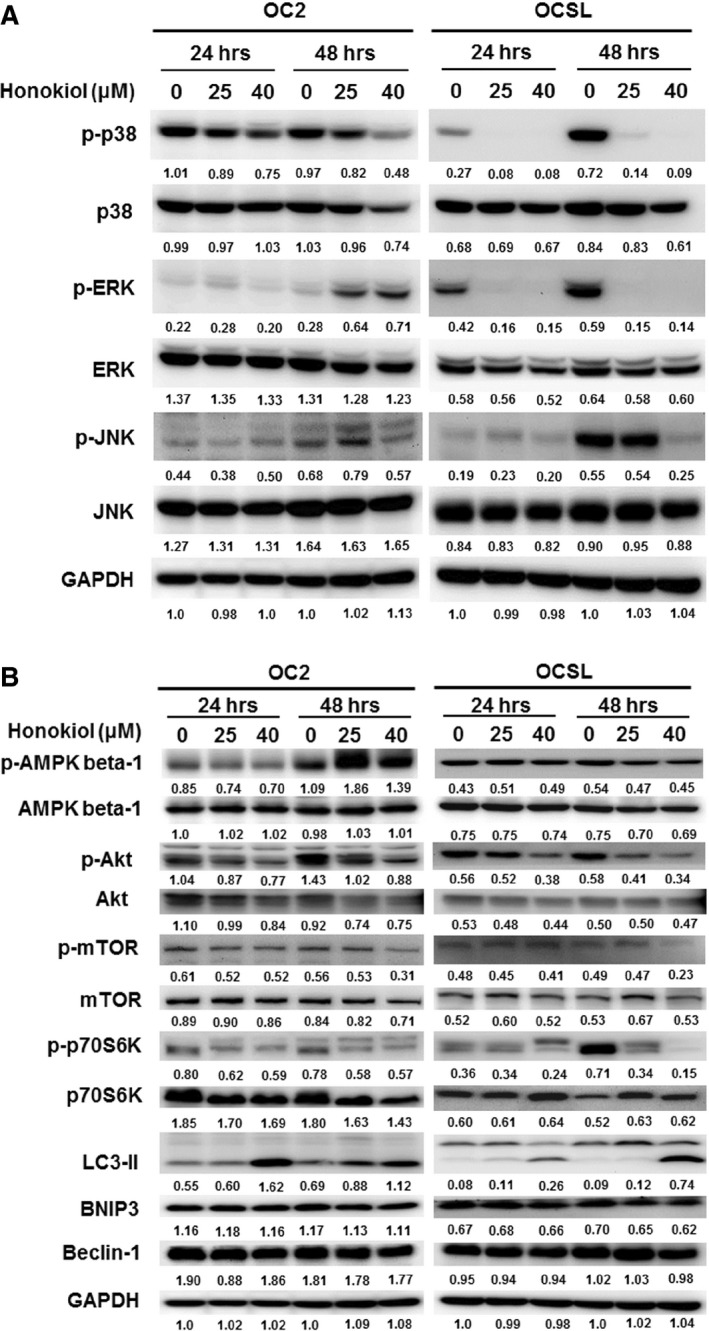
Signalling pathways modulation by honokiol. After treatment with honokiol, cells were harvested and analysed by Western blotting with antibodies against (**A**) p38, ERK and JNK, and (**B**) AMPK, Akt, mTOR, p70S6K, LC3, BNIP3 and Beclin‐1. GAPDH was used as an internal control.

**Figure 8:**
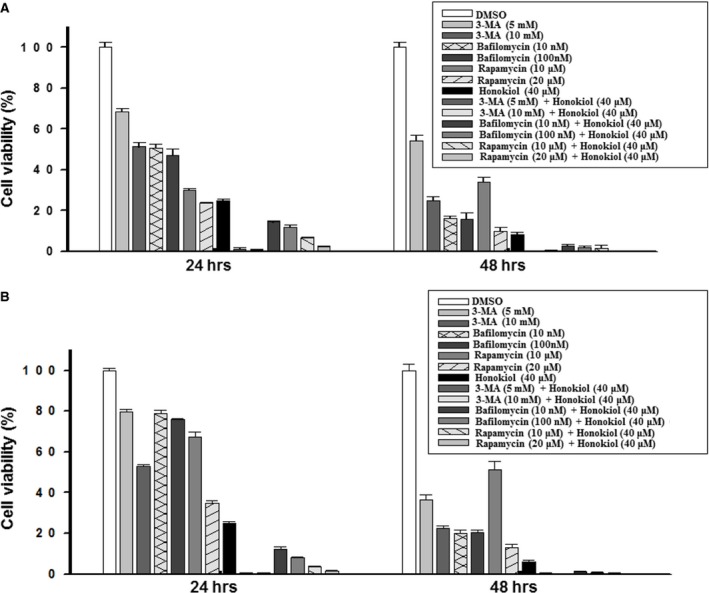
Detection of cell viability under autophagy regulation. (**A**) OC2 and (**B**) OCSL cells were treated with DMSO, 3‐MA, bafilomycin, rapamycin or honokiol, for 24 and 48 hr, and the cellular viability was measured by CCK‐8 analysis. The data present as the mean ± S.D. of three independent experiments.

The effect of honokiol on growth inhibition of OSCC cells revealed that OCSL cells are more sensitive than OC2 cells to the treatment. This might be partly due to the fact that signalling transduction in these two cell lines was different. Upon honokiol treatment, the expression levels of survival signalling (MAPKs pathway activations including p‐p38, p‐ERK and p‐JNK) in OCSL cells were notably down‐regulated, while these molecules at least p‐ERK and p‐JNK were up‐regulated in OC2 cells (Fig. 7A). In addition, the basal levels of LC3‐II molecules in these two cells were also varied, which may reflected the true that signalling in autophagy induced by honokiol in OC2 and OCSL may differ in some extents. In OCSL cells, honokiol reduced the expression of phospho‐Akt as well as the downstream molecules, such as phospho‐mTOR complex 1 (p‐mTORC1) and phosphor‐p70S6K at 24 and 48 hr (Fig. 7B). However, there were no effects on the expressions of phospho‐AMPK, BNIP3 and Beclin‐1, which were thought to be other regulators of autophagy (Fig. 7B). On the contrary, the AKT/mTOR pathway was also affected by honokiol in OC2 cells, but AMPK not BNIP3 and Beclin‐1 was activated (Fig. 7B). These results indicated that honokiol‐induced autophagy may be through down‐regulating Akt/mTORC1 pathway and/or activating the AMPK signalling in various human OSCC cells. In fact, the role of honokiol‐induced autophagy in suppressing cell viability remains unknown. Suppression of autophagy by antagonists, 3‐MA, in honokiol‐treated cells fully decreased cell viability and so did bafilomycin but to a less extent (Fig. 8). However, enhancement of autophagy by rapamycin in combination with honokiol treatment also decreased the cell viability as compared to honokiol treatment alone (Fig. 8). Thus, autophagy may play a ‘two‐edged sword’ role in honokiol treatment; that is, basal activity of autophagy could protect cells from stress, while overactivation may cause cell death as well. The cell morphology and the autophagy induced by honokiol combined with autophagy agonist, rapamycin and antagonists, 3‐MA and bafilomycin, were supplemented in Figs. [Link], [Link].

### Honokiol shows synergistic effect with chemotherapy

**Figure 9:**
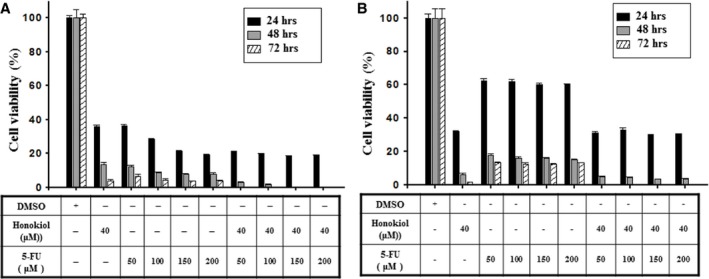
Combinatory inhibition of honokiol and 5‐FU on cell proliferation. The cellular viability of (**A**) OC2 and (**B**) OCSL cells treated with DMSO, honokiol and 5‐FU for 24, 48 and 72 hr was measured by CCK‐8 analysis. The data present as the mean ± S.D. of three independent experiments.

The effective treatments for patients with oral cancer of stages III and IV are generally surgical excision and radiation combined with chemotherapy using 5‐FU and cisplatin. Here, treatment with honokiol or 5‐FU showed a similar antiproliferation effect on OC2 cells (Fig. 9A). However, in OCSL cells, a more aggressive carcinoma, honokiol revealed a better capability than 5‐FU in suppressing cell proliferation (Fig. 9B). In addition, honokiol combined with 5‐FU exerted synergistic effect in killing OC2 cells (Fig. 9A), while it did not show such effect to suppress OCSL cells as honokiol alone was not potent enough (Fig. 9B). These results were in accord with that OCSL cells that are more sensitive than OC2 cells to honokiol treatment (Figs 1, 3 and 6).

### Honokiol exerts *in vivo* antitumour activity

**Figure 10:**
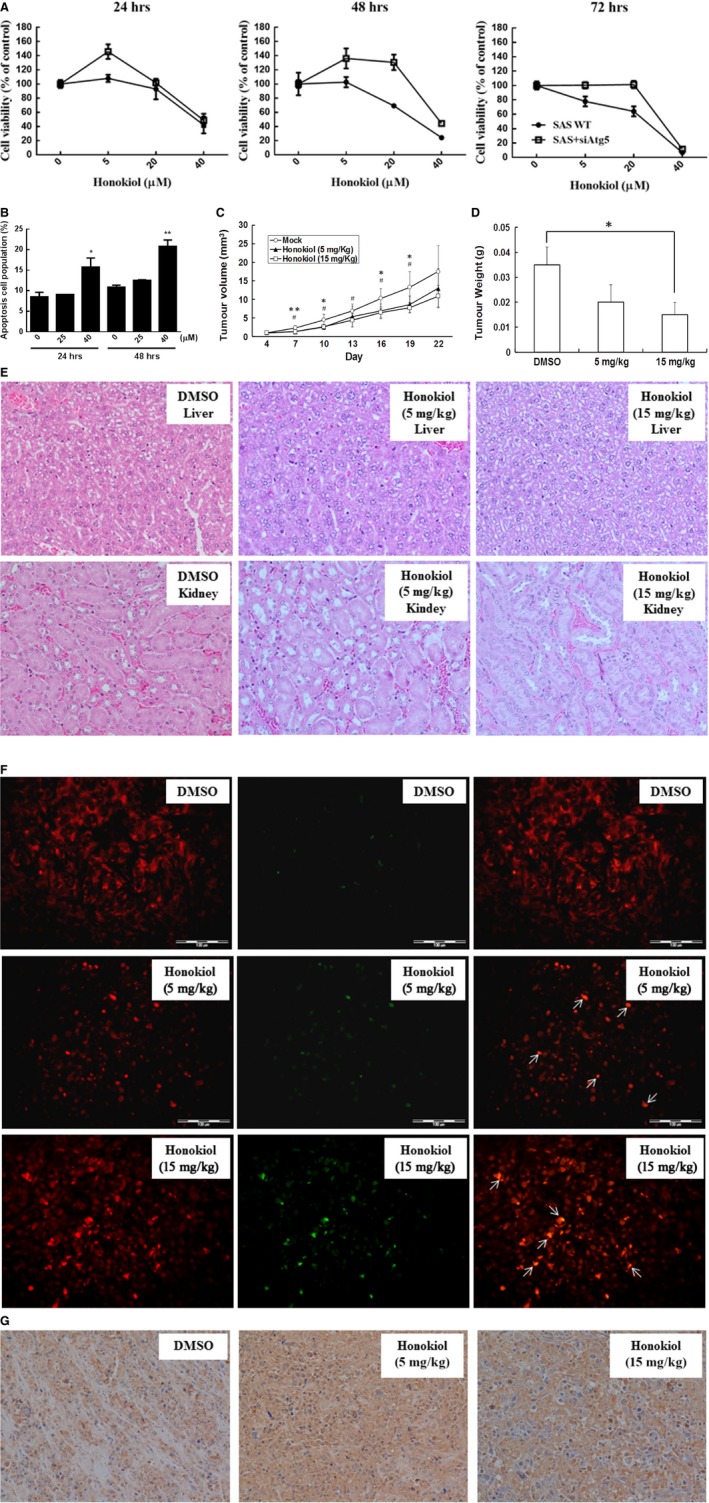
Honokiol suppresses tumour progression in nude mice. (**A**) The cell viability and (**B**) cellular apoptosis of SAS cells were evaluated under honokiol treatment. (**C**) Subcutaneous tumours derived from SAS cells were treated with or without honokiol as described. Tumour volumes were evaluated as the mean of at least three mice per group. *: honokiol 5 mg/kg *versus* DMSO; #: honokiol 15 mg/kg *versus* DMSO. (**D**) Inhibitory effects of honokiol on xenograft tumour weight. *P < 0.05, **P < 0.01 compared with DMSO. (**E**) Haematoxylin and eosin staining in liver and kidney specimens of DMSO and honokiol‐treated mouse (Magnification, ×100 and ×200). (**F**) Apoptosis induction in tumour sections with TUNEL staining. The arrow indicates the colocalization of apoptosis‐induced nuclear DNA fragmentation. (Scale bar, 20 μm) (**G**) Autophagy elevation in tumour sections were determined by immunohistochemical staining (Magnification, ×200).

To confirm the anticancer behaviour of honokiol *in vivo*, a xenograft nude mice model was established. As OC2 and OCSL cells failed to grow in nude mice, a xenograft with another OSCC cells, SAS cell, was used to evaluate the antitumour effect of honokiol *in vivo*. The antitumour effects of honokiol on SAS cells were evaluated. It was shown that honokiol suppressed SAS cell growth in a dosage‐dependent manner and genetically knock‐down of Atg5 rendered the cells less sensitive to honokiol treatment (Fig. 10A), suggesting that autophagy in the low‐dose range might potentiate honokiol‐mediated cell viability decrease. However, this phenomenon was disappeared in the group treated with high dosage level (Fig. 10A). In addition, honokiol‐mediated apoptosis was also demonstrated in SAS cells (Fig. 10B). Nude mice implanted subcutaneously with SAS xenograft for 10 days were orally administered twice a week with honokiol at the doses of 5 and 15 mg/kg. The reductions in tumour growth and the tumour volume were 29% and 40% in groups treated with 5 and 15 mg/kg, respectively, of honokiol after 3 weeks in comparison with the control group (Fig. 10C). The reduction in tumour mass after 35 days in groups treated with 5 mg/kg honokiol was 41% and that in 15 mg/kg dose was 56% (Fig. 10D). The animals demonstrated general tolerance with honokiol treatment in this setting, and no significant loss of the body weight (data not showed) and toxicity of liver and kidney (Fig. 10E) were observed. Moreover, histology analysis demonstrated an apoptosis and autophagy induction in the honokiol‐treated tumour mass (Fig. 10F and G), suggesting an effective antitumour activity of honokiol *in vivo*. Collectively, we demonstrated that honokiol could be a new candidate to be used in oral cancer therapy.

## Discussions

Human OSCC is an aggressive cancer, and the incidence rate has been gradually increasing in past 10 years. Although surgery and radiation treatments in combination with chemicals are the major therapeutic strategies for OSCC patients, the overall 5‐year death rate is reaching 50% 26. Betel nut extract has been demonstrated to be able to promote oral carcinogenesis, tumour migration and early invasion 27, 28. In this study, two OSCC cell lines, OC2 and OCSL, which are established from two Taiwanese male patients with habit of betel quit chewing, were used to evaluate the anticancer behaviour of honokiol. Among them, OCSL cells show more aggressive phenotype than OC2 cells 25.

Honokiol, a biphenolic phytochemical, is isolated from the bark of the traditional Chinese medicine *Magnolia officinalis*. It has been shown to exert multiple biological behaviours, including analgesic, anxiolytic, neuroprotection, antispasmodic, antidepressant, antimicrobial, antithrombotic, anti‐inflammatory, and antitumorigenic properties 11, 12, 29, 30, 31, 32, 33, 34, 35, 36. Arrestingly, honokiol has been reported to induce apoptosis and/or growth inhibition in various cancers including lung, breast, colon and prostate cancer *in vitro* and/or *in vivo* 10, 11, 20, 22, 37, 38. In the present study, we conclude that honokiol exerted multiple actions against these two cell lines by arresting cell cycle at G1 phase, inducing apoptosis, regulating cell signalling pathway and, in particular, provoking autophagy. One of the mechanisms for growth suppression was that honokiol retards cell cycle at G1 stage, which was demonstrated by the decreased expression of cyclin D1 and cyclin‐dependent kinases (Cdk2 and Cdk4), and caused an increase in Cdk inhibitors, p21 and p27 (Fig. 2B and D). This finding is consistent with the reports in malignant pleural mesothelioma, melanoma, T cell leukaemia, non‐small‐cell lung cancer, skin cancer, pancreatic cancer, colorectal cancer, breast cancer and prostate cancer 39, 40, 41, 42, 43, 44, 45, 46, 47.

In addition, apoptosis activation also showed a contribution in the honokiol‐mediated antiproliferation in human OSCC cells (Fig. 3). Although the apoptosis elevated by honokiol was not found in the early stage (at 24 hr), the apoptotic cells was observed notably at extended treatment (at 48 hr; Fig. 3). The heterogeneity of OSCC cell origins may vary in response to honokiol treatment. For example, an intrinsic pathway of caspase‐dependent apoptosis was found in the OC2 cells, and both of intrinsic and extrinsic pathways of caspase‐dependent apoptosis were found in the OCSL cells (Fig. 4). This result demonstrated that honokiol could induce apoptosis in human OSCC cells through multiple pathways. Honokiol‐induced cell growth inhibition in OC2 and OCSL cells was 92% (40 μM) and 85% (30 μM) at 48‐hr treatment (Fig. 1). However, the apoptotic cells in these settings accounted for 12% and 12.3%, respectively (Fig. 3). These data suggest that honokiol‐mediated apoptosis participated partially in anticancer benefit in human OSCC cells.

In addition to apoptosis, other cell phenotypes of OSCC cells induced by honokiol were also evaluated. Autophagy, a catabolic process by recycling cellular components and damaged organelles in response to environmental stress, was induced as the autophagic biomarker, LC3‐II, was elevated and could be found in the honokiol‐treated OC2 and OCSL cells (Fig. 5). The data were consistent with the recent finding in honokiol‐treated human glioblastoma multi‐forme cells 48. Furthermore, LC3 aggregation, a characteristic of autophagosome formation, was observed in rapamycin‐ or honokiol‐treated groups (Fig. 6), and the degradation of p62 with increasing dosage of honokiol in OC2 and partially in OCSL cells was evident, suggesting an autophagic flux was proceeded. Here, we demonstrated that autophagy and autophagic flux were induced by honokiol in human OSCC cells (Figs 5 and 6).

Honokiol has been described to regulate multiple signalling pathways, molecular and cellular targets including nuclear factor‐κB (NF‐κB), STAT3, epidermal growth factor receptor (EGFR), c‐Met, c‐FLIP, bone morphogenetic protein 7 (BMP7), p53, RhoA, cell survival signalling and cell cycle mediators 11, 49, 50, 51, 52. To identify the mechanisms involved in honokiol‐mediated growth inhibition of OSCC cells, three survival signalling including p38, Erk and JNK pathways and four autophagy‐related signalling pathways including AMPK, Akt/mTOR, BNIP3 and Beclin‐1 were examined. Figure 7A shows that honokiol could trigger various signalling pathways in multiple OSCC cells. Suppression of Akt/mTOR/p70S6K signalling pathway was observed in both honokiol‐treated OC2 and OCSL cells (Fig. 7B), and the AMPK signalling was also triggered by honokiol in OC2 cells (Fig. 7B). These data indicated that Akt/mTOR (a key factor) and/or AMPK pathways may be responsible for the induction of autophagy in honokiol‐treated human OSCC cells. In addition, it has been reported that honokiol inhibits anti‐apoptotic gene products including cIAP1/2, Bcl‐2, c‐FLIP and survivin through the down‐regulation of Akt‐ and NF‐κB‐related pathways 52, 53. Suppression of Akt/mTOR pathway was also reported to alter autophagy activation in human thyroid cancer cells 54. Therefore, the results of OSCC cells treated with honokiol were in line with others, and honokiol‐mediated apoptosis and/or autophagy in human OSCCs may involve the down‐regulation of Akt and related anti‐apoptotic or anti‐autophagic factors.

Autophagy is a catabolic process involving cellular recycling and is believed to help in survival and longevity of cancer cells by buffering metabolic stress. Inhibition of autophagy in an environment of nutrient deprivation can induce cell death. However, enhancement of autophagy can also be a beneficial tool in the fight against cancers 55. In order to evaluate the role of autophagy induced by honokiol in decreasing the cellular viability of OSCC cells, rapamycin, 3‐MA or bafilomycin was used to enhance or block autophagy and/or autophagic degradation. Modulation of autophagy by chemicals has no rescuing effect on decreased cell viability caused by honokiol (Fig. 8). However, genetically knock‐down of Atg5 by siRNA rendered SAS cells less sensitive to honokiol treatment in low‐dose range, and this protection effect was not sustained under high dosage treatment (Fig. 10A). This phenomenon was consistent with previous finding 55, and the resultant low cell viability induced by honokiol was partially due to autophagy induction. The modulation of autophagy in honokiol treatment by different interventions, that is chemicals or siRNA, may lead to different interpretations. It was agreed that autophagy may play a ‘two‐edged sword’ role in many cases. Autophagy has also been reported to delay the apoptotic death in human breast cancer cells under camptothecin‐induced DNA damage 56. A well‐known molecule, p53, has been demonstrated to be a master regulator of autophagy and apoptosis 57. Mutations of p53 gene were observed in human OSCC cells, including these cell lines, OC2, OCSL and SAS cells 25, 58. The complex of p53/HMGB1 has been reported to cross‐regulate autophagy and apoptosis in human colorectal cancer cells 59. Loss of p53 increases cytoplasmic HMGB1 levels and facilitates cell survival through autophagy activation. Comparatively, loss of HMGB1 elevates cytoplasmic p53 levels and the p53‐mediated apoptosis 59. Another molecule, Bcl‐2, an anti‐apoptotic factor, also has been reported to regulate apoptosis as well as autophagy through interaction with Beclin‐1 and/or ATG12 57. Here, our results provided the clue that autophagy may potentiate honokiol‐mediated multiple effects, at least on decreasing cell viability, and the interplay between apoptosis and autophagy in honokiol‐treated human OSCC cells needs further investigation.

Importantly, honokiol shows better antiproliferation effect than 5‐FU and has a synergistic effect with 5‐FU in the treatment of human OSCC cells (Fig. 9). In the current reports, honokiol has been demonstrated to augment and exhibit a synergism with chemotherapeutic agents including rapamycin, gemcitabine, fluconazole and imatinib 20, 44, 60, 61. Moreover, in animal models, our study and others show no obvious impacts in pathologic changes and genotoxicity after honokiol treatment, suggesting that honokiol is safe in animals and thus could be applicable in clinical practice^10^, ^62^, ^63^, ^64^. Our results indicated that honokiol either alone or in combination with other drugs has great potential for human OSCCs therapeutics.

Huang *et al*. have identified a side population (SP) cells harbouring stem cell‐like properties in the eight human OSCC cell lines 65. Among these cell lines, SAS shows more tumorigenic potential in xenografts and displays more SP cells 65. In the present study, OC2 and OCSL cells show non‐tumorigenicity *in vivo*; however, SAS cells display tumorigenic capability in xenograft mice model. It was suggested that SP cells might be the absence or few in the population of OC2 and OCSL cells. In addition, preclinical researches of honokiol have showed wide‐ranging biological and clinically relevant effects on tumour treatment, without appreciable toxicity in animal models 10, 34, 66, 67. These effects include promoting apoptosis, arresting cell cycle, making direct cytotoxicity, down‐regulating cancer cell signalling pathway, regulating genetic expression, inhibiting angiogenesis, enhancing synergistic effects with chemotherapeutic agents and radiation and suppressing multidrug resistance 10, 66, 67. The toxicological profile of honokiol is promising. Accordingly, Wang *et al*. had revealed that no significant abnormalities are observed in rats, including body weight, mean daily food intake, haematological values, serum biochemical values and tissue pathologic changes, in administration with high dosage of honokiol (80 mg/kg) 62. Therefore, honokiol appears to be a promising natural agent for cancer prevention and therapy, and most importantly, it is proven to be safety *in vivo*.

In the present study, we found that both OC2 and OCSL cells cannot establish tumours in nude mice; hence, SAS cells, another OSCC cell line, were used to evaluate the antitumour benefit of honokiol *in vivo*. In this study, honokiol has shown the promising antitumour activity without exacerbating significant toxicities with regard to the body weight loss and the liver and kidney histological examinations, which was in line with other observations, previously 10, 21, 22, 62, 63, 64. Altogether, our study indicates that honokiol is a promising candidate to develop for the prevention and treatment of human OSCCs.

## Conflict of interest

We have read the Journal's policy, and the authors confirm that there is no conflict of interests.

## Author contributions

CHK, KJH and YRL conceived and designed the experiments; CHK, SHC, CYL and YRL analysed the data; CHK, KJH and YRL wrote the first draft of the manuscript; CHK, KJH, SHC, CYL and YRL read and confirm the criteria for authorship.

## Supporting information


**Fig. S1** Honokiol inhibited the growth of human normal cells. Hs68 cells were incubated with various concentration of honokiol, and the cell viability was determined by CCK‐8 analysis. The data present as the mean ± S.D. of three independent experiments

**Fig. S2** (**A**) The OC2 cell morphology and the autophagy induced by honokiol combined with autophagy agonist, rapamycin, and antagonists, 3‐MA and bafilomycin. The morphologic changes were observed under a microscope. (**B**) OC2 cell incubated with DMSO, 3‐MA, bafilomycin, rapamycin and honokiol, and then the cell lysates were collected for western blotting of LC3‐II and p62 proteins. GAPDH was used as an internal control

**Fig. S3** (**A**) The OCSL cell morphology and the autophagy induced by honokiol combined with autophagy agonist. The morphologic changes were observed under a microscope. (**B**) OCSL cell incubated with DMSO, 3‐MA, bafilomycin, rapamycin and honokiol, and then the cell lysates were collected for western blotting of LC3‐II and p62 proteins. GAPDH was used as an internal control
