# Supplementary figures and images for: Honokiol inhibits in vitro and in vivo growth of oral squamous cell carcinoma through induction of apoptosis, cell cycle arrest and autophagy
Source: J Cell Mol Med. 2018 Jan 24;22(3):1894–908. doi: 10.1111/jcmm.13474 (PMC5824386; doi:10.1111/jcmm.13474)

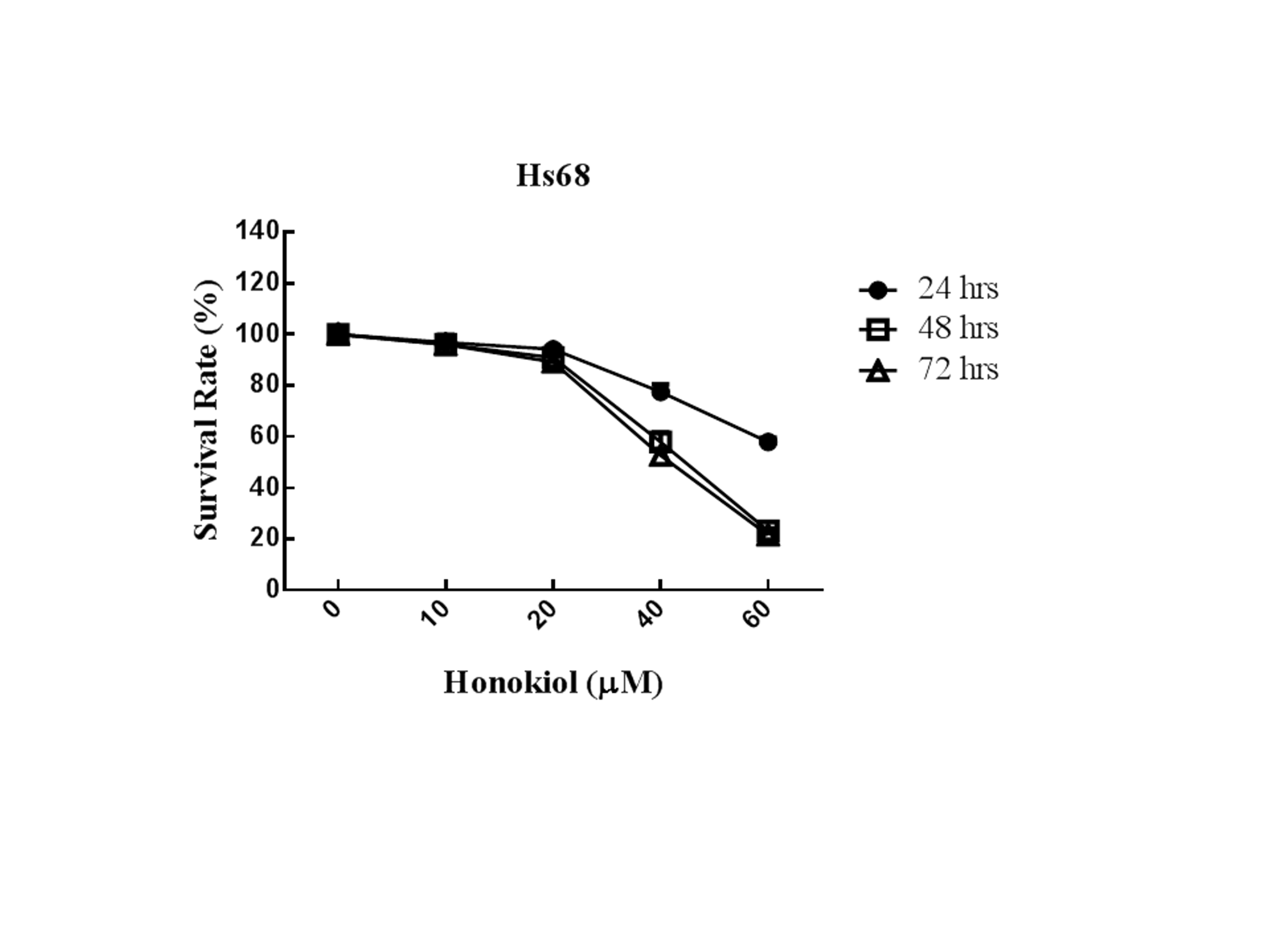

Supplement: Supplementary file 1 — Fig. S1 Honokiol inhibited the growth of human normal cells. Hs68 cells were incubated with various concentration of honokiol, and the cell viability was determined by CCK‐8 analysis. The data present as the mean ± S.D. of three independent experiments [file JCMM-22-1894-s001.tiff]

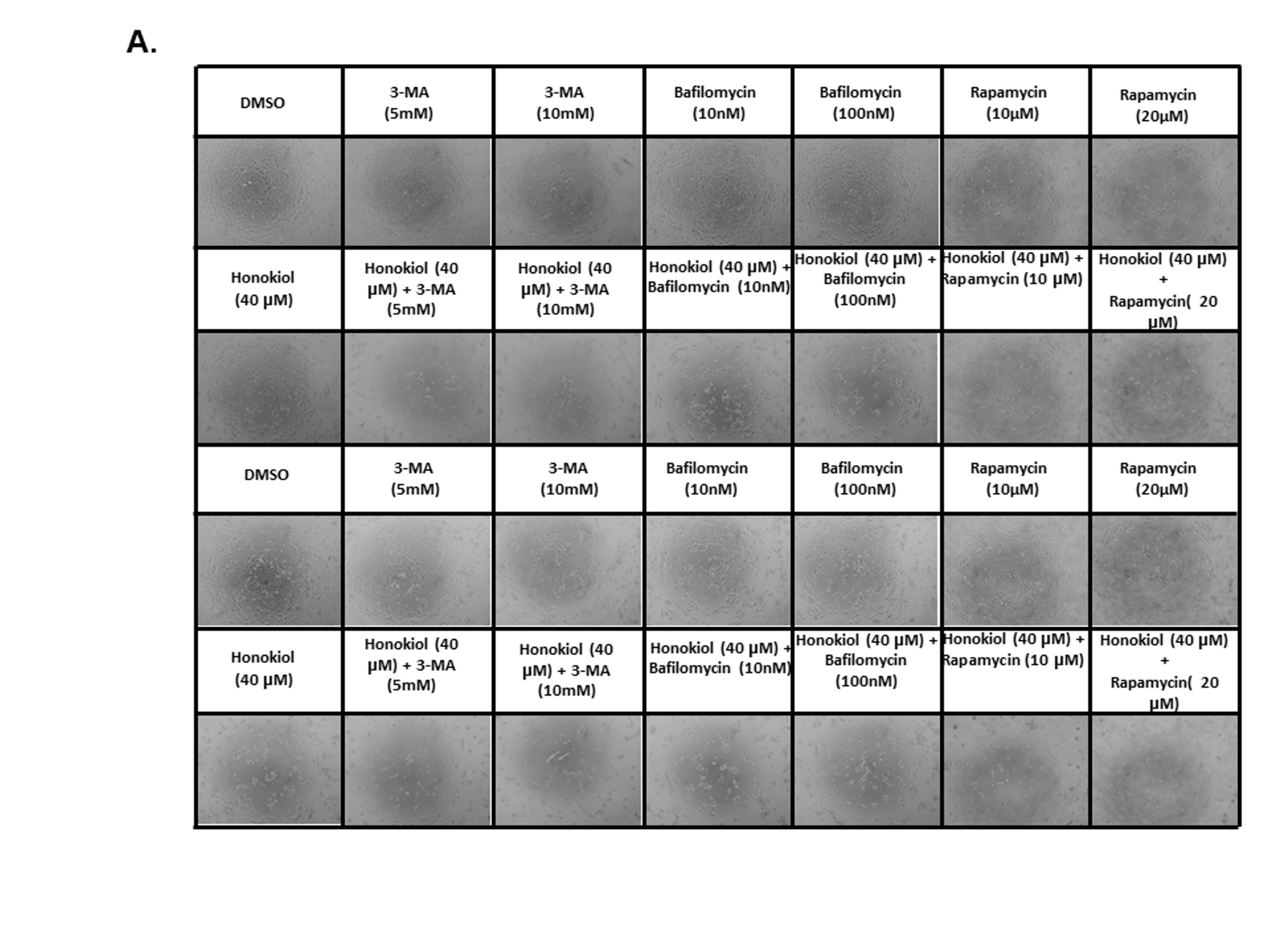

Supplement: Supplementary file 2 — Fig. S2 (A) The OC2 cell morphology and the autophagy induced by honokiol combined with autophagy agonist, rapamycin, and antagonists, 3‐MA and bafilomycin. The morphologic changes were observed under a microscope. (B) OC2 cell incubated with DMSO, 3‐MA, bafilomycin, rapamycin and honokiol, and then the cell lysates were collected for western blotting of LC3‐II and p62 proteins. GAPDH was used as an internal control [file JCMM-22-1894-s002.tiff]

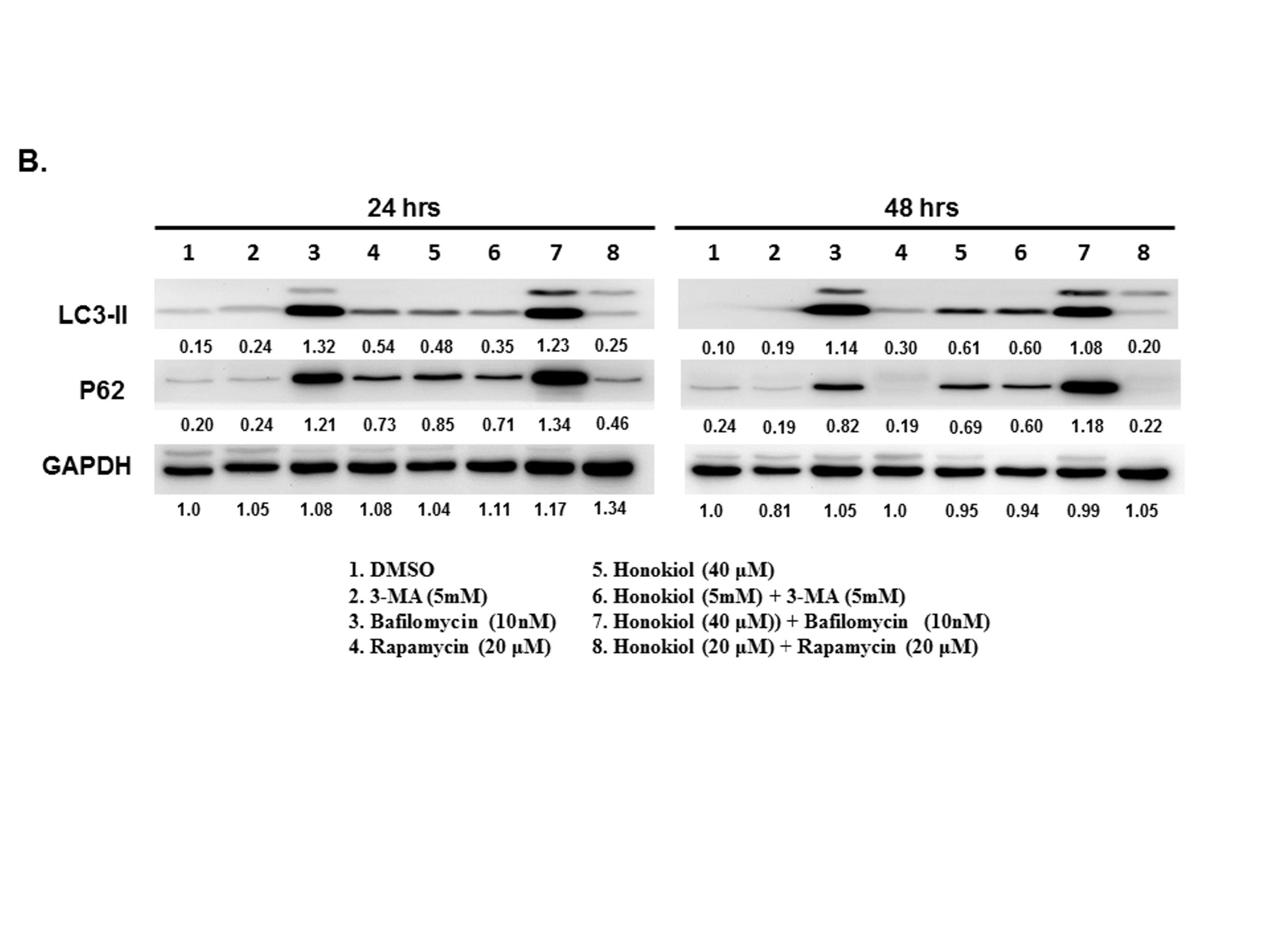

Supplement: Supplementary file 3 [file JCMM-22-1894-s003.tiff]

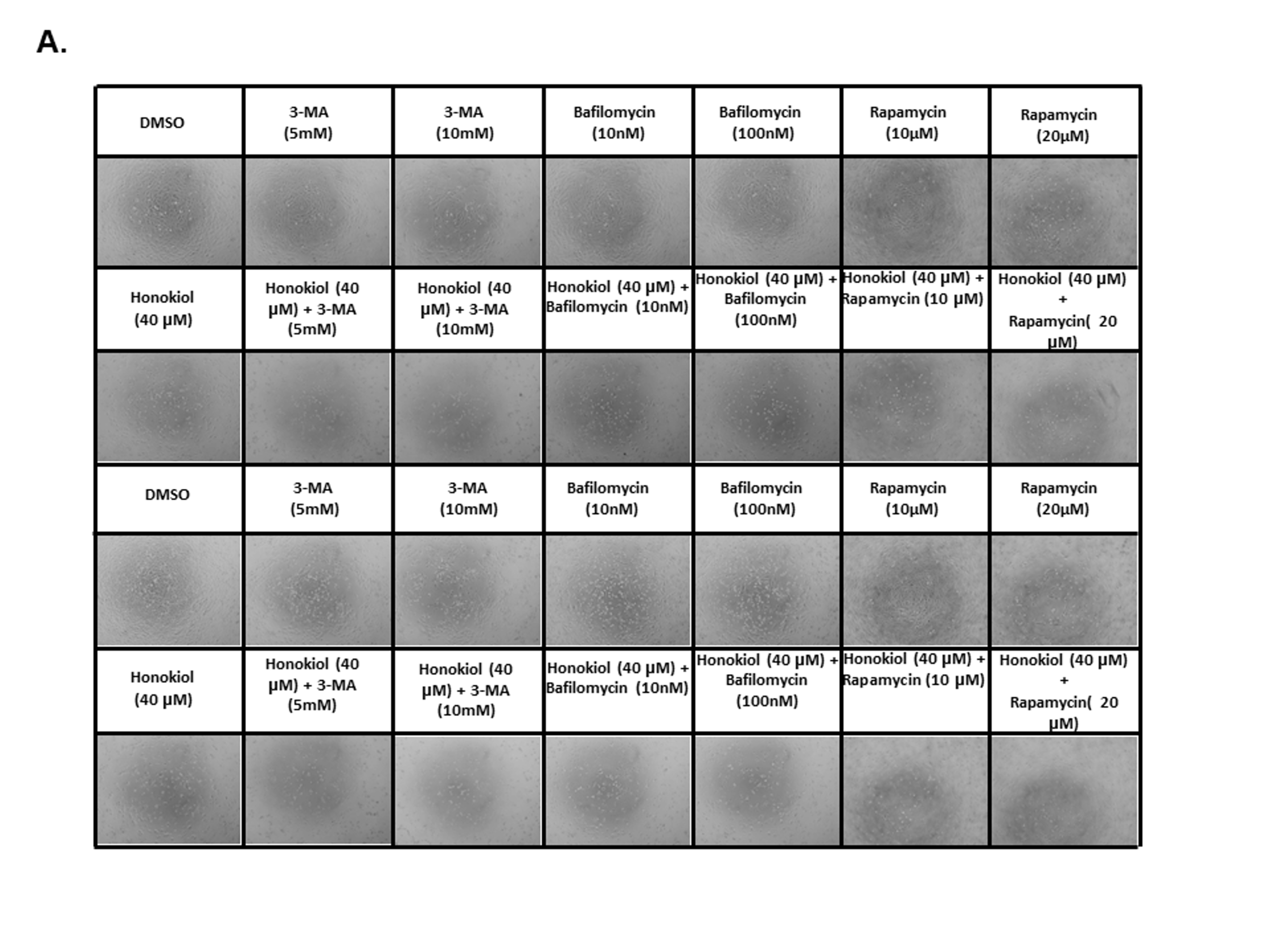

Supplement: Supplementary file 4 — Fig. S3 (A) The OCSL cell morphology and the autophagy induced by honokiol combined with autophagy agonist. The morphologic changes were observed under a microscope. (B) OCSL cell incubated with DMSO, 3‐MA, bafilomycin, rapamycin and honokiol, and then the cell lysates were collected for western blotting of LC3‐II and p62 proteins. GAPDH was used as an internal control [file JCMM-22-1894-s004.tiff]

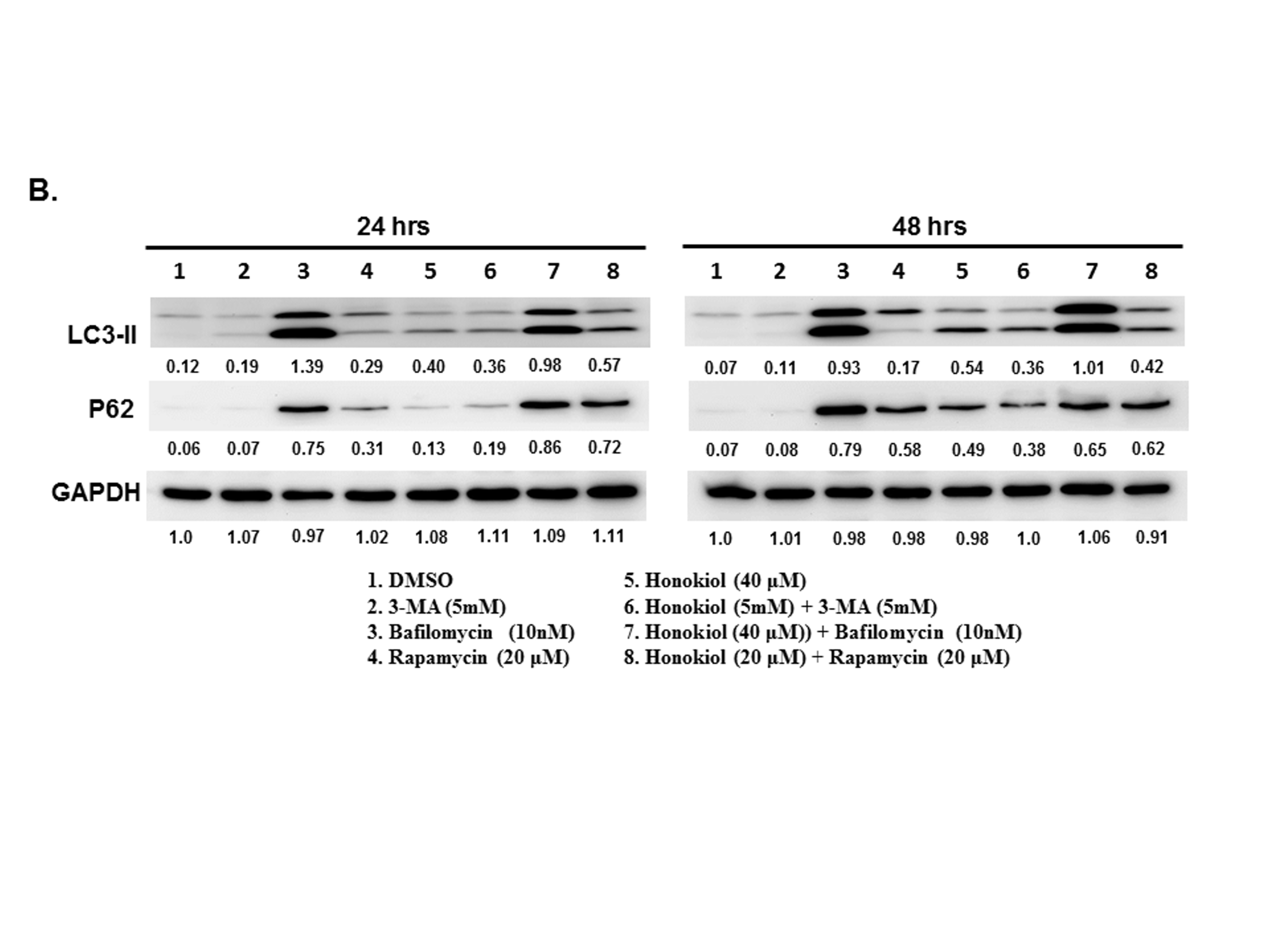

Supplement: Supplementary file 5 [file JCMM-22-1894-s005.tiff]
